# The Essential Role of Cholesterol Metabolism in the Intracellular Survival of Mycobacterium leprae Is Not Coupled to Central Carbon Metabolism and Energy Production

**DOI:** 10.1128/JB.00625-15

**Published:** 2015-10-28

**Authors:** Maria Angela M. Marques, Marcia Berrêdo-Pinho, Thabatta L. S. A. Rosa, Venugopal Pujari, Robertha M. R. Lemes, Leticia M. S. Lery, Carlos Adriano M. Silva, Ana Carolina R. Guimarães, Georgia C. Atella, William H. Wheat, Patrick J. Brennan, Dean C. Crick, John T. Belisle, Maria Cristina V. Pessolani

**Affiliations:** a Laboratório de Microbiologia Celular, Instituto Oswaldo Cruz, Fundação Oswaldo Cruz, Rio de Janeiro, RJ, Brazil; b Department of Microbiology, Immunology and Pathology, Colorado State University, Fort Collins, Colorado, USA; c Laboratório de Genômica Funcional e Bioinformática, Instituto Oswaldo Cruz, Fundação Oswaldo Cruz, Rio de Janeiro, RJ, Brazil; d Laboratório de Bioquímica de Lipídeos e Lipoproteínas, Instituto de Bioquímica Médica, Universidade Federal do Rio de Janeiro, Rio de Janeiro, RJ, Brazil

## Abstract

Mycobacterium leprae induces the formation of lipid droplets, which are recruited to pathogen-containing phagosomes in infected macrophages and Schwann cells. Cholesterol is among the lipids with increased abundance in M. leprae-infected cells, and intracellular survival relies on cholesterol accumulation. The present study investigated the capacity of M. leprae to acquire and metabolize cholesterol. *In silico* analyses showed that oxidation of cholesterol to cholest-4-en-3-one (cholestenone), the first step of cholesterol degradation catalyzed by the enzyme 3β-hydroxysteroid dehydrogenase (3β-HSD), is apparently the only portion of the cholesterol catabolic pathway seen in Mycobacterium tuberculosis preserved by M. leprae. Incubation of bacteria with radiolabeled cholesterol confirmed the *in silico* predictions. Radiorespirometry and lipid analyses performed after incubating M. leprae with [4-^14^C]cholesterol or [26-^14^C]cholesterol showed the inability of this pathogen to metabolize the sterol rings or the side chain of cholesterol as a source of energy and carbon. However, the bacteria avidly incorporated cholesterol and, as expected, converted it to cholestenone both *in vitro* and *in vivo*. Our data indicate that M. leprae has lost the capacity to degrade and utilize cholesterol as a nutritional source but retains the enzyme responsible for its oxidation to cholestenone. Thus, the essential role of cholesterol metabolism in the intracellular survival of M. leprae is uncoupled from central carbon metabolism and energy production. Further elucidation of cholesterol metabolism in the host cell during M. leprae infection will establish the mechanism by which this lipid supports M. leprae intracellular survival and will open new avenues for novel leprosy therapies.

**IMPORTANCE** Our study focused on the obligate intracellular pathogen Mycobacterium leprae and its capacity to metabolize cholesterol. The data make an important contribution for those interested in understanding the mechanisms of mycobacterial pathogenesis, since they indicate that the essential role of cholesterol for M. leprae intracellular survival does not rely on its utilization as a nutritional source. Our findings reinforce the complexity of cholesterol's role in sustaining M. leprae infection. Further elucidation of cholesterol metabolism in the host cell during M. leprae infection will establish the mechanism by which this lipid supports M. leprae intracellular survival and will open new avenues for novel leprosy therapies.

## INTRODUCTION

Leprosy is a granulomatous disease caused by Mycobacterium leprae, an obligate intracellular organism that affects mainly the skin and peripheral nerves. This ancient disease, which can be considered one of the oldest human infections (1), is still a significant public health problem in developing countries, including India, Brazil, and Indonesia, which collectively account for 83% of all new cases worldwide (2). Although the global prevalence of leprosy has declined from 5.2 million in the 1980s to approximately 200,000 today following the implementation of multidrug therapy (MDT) (2), the number of new cases of leprosy in regions where the disease is endemic is an indication that the transmission of M. leprae is still an issue. Moreover, at least in Brazil, the prevalence of undiagnosed cases in areas with a high burden of disease is reported to be much higher than the registered prevalence (3). MDT is effective in bacterial killing but does not prevent reactional episodes, the major cause of nerve injury and physical disabilities seen in individuals affected by the disease (4). Thus, novel drug combinations are needed for a better management of leprosy patients and prevention of physical sequelae.

The leprosy bacillus is an obligate intracellular pathogen with preferential tropism for macrophages of the skin and Schwann cells of the peripheral nerves. The inability of M. leprae to grow extracellularly or in axenic medium can be explained by the reduced coding capacity of its degenerate genome (1,605 functional genes and 1,115 pseudogenes) (5). These characteristics and a lack of experimental models of disease have hampered the study of host-pathogen interactions in leprosy. Nevertheless, insight into the pathogenesis of leprosy can be gained through the use of *in vitro* models of infection that allow dissection of host cell-pathogen interactions and complementation with *ex vivo* data based on analyses of clinical samples from leprosy patients.

As early as 1863, Virchow observed the leprosy bacillus residing inside foamy macrophages (called Virchow's cells) (6). With the discovery and characterization of M. leprae-derived lipids, such as phthiocerol dimycocerosate (PDIM) and phenolic glycolipid I (PGL-I), it was initially believed the lipid-heavy Virchow's cells resulted from these bacterial products (7, 8). However, recent reports demonstrate that Virchow's cells accumulate host-derived lipids (9, 10). Virchow's cells found in dermal lesions are also highly positive for adipose differentiation-related protein (ADRP), a classical cholesterol-containing lipid droplet (LD) marker. This suggests that the foamy phenotype is in part derived from the accumulation of LDs (9, 11). The ADRP marker is induced by M. leprae infection and localizes to M. leprae-containing phagosomes, indicating a close association between LDs and the pathogen-containing vacuole (11, 12). More recently, we analyzed nerve biopsy specimens and used *in vitro* cultures of Schwann cells to show that the foamy phenotype of the lepromatous leprosy (LL) nerves is also related to the capacity of M. leprae to induce LD biogenesis in this cell type (13, 14). Cholesterol was confirmed as a host lipid that accumulates in M. leprae-infected macrophages. Further, M. leprae increases *de novo* synthesis of cholesterol as well as exogenous uptake of low-density-lipoprotein (LDL) cholesterol by upregulating the expression of genes involved in these pathways (12). Of importance, cholesterol has been found to colocalize to M. leprae-containing phagosomes, and impairment of cholesterol metabolism significantly decreased intracellular bacterial survival both *in vitro* and *in vivo* (12, 15).

The dependence of mycobacteria on host lipid molecules for successful infection and persistence has been extensively analyzed in the context of Mycobacterium tuberculosis. M. tuberculosis also mediates formation of foamy cells, a process apparently critical for bacterial persistence in the host (16, 17). Additionally, a large body of literature describing the importance of cholesterol for persistence of M. tuberculosis in the host has accumulated (18, 19). M. tuberculosis possesses the ability to degrade and use cholesterol as an energy source and for the biosynthesis of mycobacterial lipids (18). This metabolic capacity for cholesterol utilization appears to be particularly important during the latent phase of M. tuberculosis infection, when other carbon sources become limited (18, 20). The contribution of cholesterol to the *in vivo* growth of M. tuberculosis and tuberculosis pathogenesis has resulted in the elucidation of M. tuberculosis genes directly or indirectly involved in cholesterol metabolism (for a review, see reference 21).

In light of the current understanding of cholesterol catabolism in M. tuberculosis, and in the face of the essential role of cholesterol for intracellular M. leprae survival, the present study revisited the M. leprae genome annotated in 2001 (5), looking for orthologous enzymes/proteins of M. tuberculosis genes associated with cholesterol metabolism (21). This *in silico* analysis was complemented with investigation of the capacity of M. leprae to metabolize cholesterol. The data showed that M. leprae lost essentially all the genes associated with cholesterol catabolism in M. tuberculosis and consequently the capacity to degrade both the sterol rings and the lateral chain but retained the ability to oxidize cholesterol to cholest-4-en-3-one (cholestenone). These findings reinforce the complexity of cholesterol's role in sustaining M. leprae infection.

## MATERIALS AND METHODS

### Mycobacterial culture and growth medium.

M. leprae purified from athymic *nu/nu* mouse footpads was donated by the National Hansen's Disease Program, Laboratory Research Branch, Louisiana State University, Baton Rouge, LA, USA, and by Lauro de Sousa Lima Institute, Bauru, São Paulo, Brazil. M. tuberculosis mc^2^6230 was kindly donated by William R. Jacobs, Albert Einstein College of Medicine, Bronx, NY (22). M. tuberculosis was grown on Middlebrook 7H9 broth supplemented with 10% oleic acid-albumin-dextrose-catalase (OADC), 0.5% glycerol, 0.05% tyloxapol, 0.2% Casamino Acids, 50 μg/ml ampicillin, and 24 μg/ml pantothenate until cell growth reached log phase. Inactivation of mycobacterial cells was achieved by heating at 100°C for 15 min.

### Radiorespirometry assay.

**FIG 1:**
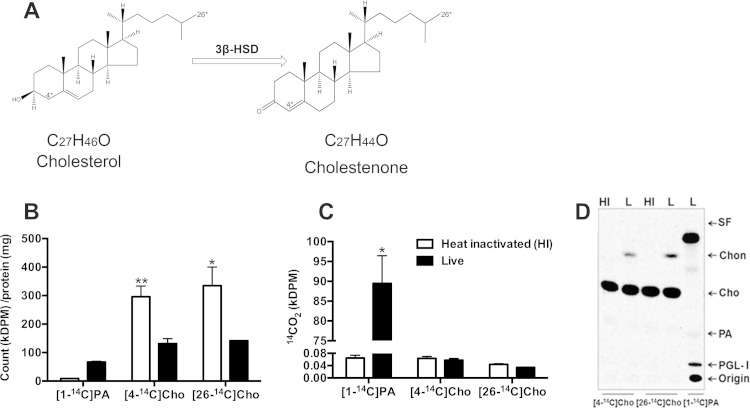
Cholesterol is incorporated into M. leprae cells but is not used as an energy or carbon source. (A) Chemical structures and reaction of the oxidation of cholesterol to cholestenone catalyzed by 3β-HSD in M. tuberculosis. Asterisks indicate the position of the radiolabel (carbon-4 and carbon-26) in the molecules. The chemical structures were designed using Chem Draw (PerkinElmer). (B to D) Heat-inactivated (HI) and live (L) M leprae (6 × 10^7^ bacteria/200 μl) were incubated for 4 days at 33°C with 1 μCi/ml [4-^14^C]cholesterol, [26-^14^C]cholesterol, or [1-^14^C]palmitic acid (PA). (B) Bacteria were washed, and incorporation of radiolabeled lipids into M. leprae cells was expressed as kilodisintegrations per minute (kDPM) per milligram of protein. (C) For the radiorespirometry assay, the ^14^CO_2_ trapped on strips was measured and expressed as DPM. Data are means and standard deviations of the results of three experiments performed in duplicate. (D) TLC of lipid extracts of M. leprae cells labeled with [4-^14^C]cholesterol, [26-^14^C]cholesterol, or [1-^14^C]palmitic acid. Labeled M. leprae cells were washed, extracted with chloroform-methanol (2:1), and resolved by TLC analysis with chloroform-methanol (95:5). Radiolabeled lipids were detected using a PhosphorImager. All data are representative of three independent experiments performed in duplicate. SF, solvent front; Chon, cholestenone; Cho, cholesterol; PA, palmitic acid; PGL-I, phenolic glycolipid I. **, *P* = 0.0013, and *, *P* < 0.05, for heat-inactivated versus live M. leprae.

M. leprae and M. tuberculosis, freshly purified from athymic *nu/nu* mouse footpads and harvested from exponential cultures, respectively, were washed three times with phosphate-buffered saline (PBS; pH 7.4) containing 0.05% tyloxapol (PBS-Ty). M. leprae was suspended in Middlebrook 7H9 broth supplemented with 0.1% casein hydrolysate, 0.5% BSA, 0.05% tyloxapol, 50 μg/ml ampicillin and 48 U/ml catalase. M. tuberculosis was suspended in Middlebrook 7H9 broth supplemented with 0.05% tyloxapol, 0.2% Casamino Acids, 50 μg/ml ampicillin, and 24 μg/ml pantothenate. The respirometry assay is based on the method of Buddemeyer (23), with minor modifications. Suspensions of mycobacterial cells (6 × 10^7^ bacteria in 200 μl) containing 1 μCi/ml of [1-^14^C]palmitic acid (specific activity, 55 mCi/mmol) (American Radiolabeled Chemicals, Inc., Saint Louis, MO), [4-^14^C]cholesterol (specific activity, 50 mCi/mmol), or [26-^14^C]cholesterol (specific activity, 52 mCi/mmol) (Quotient Bioresearch Ltd., Cardiff, United Kingdom) (see cholesterol structure in Fig. 1A) were transferred to 500-μl Eppendorf tubes without caps and placed in 6-ml scintillation vials containing two filter paper strips (40 by 8 mm) impregnated with 100 μl of 2 N NaOH. After incubation for 24 and 72 h for M. tuberculosis and 4 days for M. leprae, radiolabeled CO_2_ captured on the filter paper strips was measured by transferring them to a new scintillation vial containing 100 μl of concentrate acetic to neutralize the strips, adding 5 ml of Optima Gold (PerkinElmer, Waltham, MA) scintillation cocktail, and counting on a Beckman LS 600 liquid scintillation counter (Beckman Coulter, Pasadena, CA). Captured ^14^CO_2_ was a measure of cumulative β oxidation of palmitic acid or cholesterol.

### Cholesterol incorporation by M. leprae.

Live or heat-inactivated M. leprae cells were incubated with [1-^14^C]palmitic acid, [4-^14^C]cholesterol, or [26-^14^C]cholesterol for 4 days as described in “Radiorespirometry assay” above. M. leprae cells were collected by centrifugation at 12,000 × *g* for 8 min, and pellets were washed three times with PBS-Ty. The final bacterial cell pellet was suspended with 100 μl PBS, and an aliquot was transferred to a scintillation vial containing Optima Gold Plus scintillation cocktail and counted on a Beckman LS 600 liquid scintillation counter.

### Lipid analysis.

Mycobacterial cells labeled with [1-^14^C]palmitic acid, [4-^14^C]cholesterol, or [26-^14^C]cholesterol for various times were pelleted by centrifugation. The conditioned medium was also collected for extraction of lipids. The bacterial cells were washed three times with PBS-Ty. Bacterial cells and conditioned medium were extracted with chloroform-methanol (2:1) (24), and the extracts were dried under N_2_ after two washes (25). Radiolabeled compounds were resolved by thin-layer chromatography (TLC) using silica gel G60 TLC plates (Millipore, Temecula, CA) developed three times in chloroform-ethyl acetate (97:3) or once in chloroform-methanol (95:5) and detected using a PhosphorImager (Typhoon 9400 scanner; GE Amersham, Sunnyvale, CA). Lipid extracts were also spiked with 2 μg of authentic cholesterol or cholestenone (both from Sigma-Aldrich) and loaded on TLC plates, which were developed with chloroform-methanol-acetic acid (95:4:1) or chloroform-methanol-ammonium hydroxide (95:4:1). After the TLC plates had been to the phosphorimaging screen overnight, they were sprayed with a charring solution consisting of 5% phosphomolybdic acid in 100% ethanol or anisaldehyde solution (26) and heated to visualize the lipids.

### Sample preparation for gas chromatography-mass spectrometry (GC-MS) and GC-MS conditions.

Lipids were extracted from M. leprae and noninfected mouse footpad tissue as described by Bligh and Dyer (24), followed by a second extraction with chloroform-methanol (2:1) (25). The organic extracts were dried under N_2_ and saponified. Briefly, 3 ml of 25% alcoholic potassium hydroxide solution (25 g of potassium hydroxide and 35 ml of sterile distilled water, brought to 100 ml with 100% ethanol) was added to the lipid pellets, mixed for 1 min, and incubated at 85°C for 1 h. The saponified material was cooled to room temperature, and the sterols were extracted by the addition of 1 ml of sterile distilled water and 3 ml of *n*-heptane, followed by vigorous mixing for 3 min. The heptane layer was collected and dried under N_2_. The isolated sterols were silylated by the addition of 50 μl bis(trimethylsilyl)trifluoroacetamide (BSTFA)-trimethylchlorosilane (TMCS) (99:1) (Sigma-Aldrich) and 50 μl pyridine and incubation for 1 h at 65°C.

GC-MS for M. leprae extract was performed on a Shimadzu GCMS-QP2010 Plus system (Shimadzu, Barra Funda, SP, Brazil), using an Rtx-5MS column (5% phenyl–95% dimethylpolysiloxane; 30 m by 0.25 mm by 0.25 μm; Restex, Bellefonte, PA). The injector temperature was set at 250°C. Analytes were eluted from the column with a biphasic linear thermal gradient of 120 to 250°C at a rate of 20°C per min, followed by 250 to 280°C at a rate of 5°C per min holding at 280°C for 10 min, 280 to 330°C at a rate of 10°C per min, and holding at 330°C for 6 min. Helium was used as the carrier gas, with a linear velocity of 37.5 cm/s. The derivatized lipid extracts (5 μl) were applied to GC-MS via splitless mode injection. Electroionization was performed at −70 eV, and the quadrupole mass analyzer collected data from 30 to 500 atomic mass units (amu).

GC-MS analysis of mouse tissue extract was carried out on a Shimadzu GCMS-QP2010 Plus system, using an Agilent HP Ultra 2 column (5% phenyl–95% methylpolysiloxane; 25 m by 0.20 mm by 0.33 μm; Agilent, Barueri, SP, Brazil). The injector temperature was set at 250°C. Analytes were eluted from the column using a thermal gradient of 50 to 270°C at a rate of 18°C per min and 270 to 300°C at a rate of 1°C per min. Helium was used as the carrier gas, with a linear velocity of 33.0 cm/s. A volume of 1 μl of sample was applied to the GC-MS. Electroionization was performed at −70 eV, and the quadrupole mass analyzer collected data from 40 to 600 amu.

To confirm chromatogram peak identities, retention indices were used. A steroid standards mix (0.5 μg/μl), composed of cholesterol, 7-dehydrocholesterol, ergosterol, cholestenone, α-ergostenol, stigmasterol, γ-ergostenol, lanosterol (Sigma), zymosterol, and β-sitosterol (Avanti Polar Lipids, Alabaster, AL), was processed in parallel with the sample. Additionally, the products detected by GC-MS were identified by comparing their mass spectra with those of the NIST library (NIST05).

### *In silico* analysis.

The M. leprae TN genome (accession number AL450380, updated 6 February 2015) and M. tuberculosis H37Rv genome (accession number AL123456, updated 13 June 2013) were represented using Artemis software release 16.0.0 (Sanger Institute). M. tuberculosis cholesterol-related proteins (reviewed in reference 21) were searched against the M. leprae genome using the online NCBI BLASTP tool. Proteins identified by BLAST as having more than 70% similarity and 70% coverage were considered potential matches. Those bidirectional best-hit matches were carefully examined for the presence of functional domains (Pfam and Interpro databases) that could support their involvement in cholesterol transport, conversion, or metabolism.

M. leprae putative ChoD (ML0389) and putative 3β-hydroxysteroid dehydrogenase (3β-HSD) (ML1942), M. tuberculosis putative ChoD (Rv3409c) and 3β-HSD (Rv1106c), and Streptomyces sp. 3β-HSD (Protein Data Bank [PDB] entry 1B4V) sequences were analyzed using bioinformatics tools for protein sequence annotation. Sequence similarity searches in the nonredundant and PDB databases were performed using online NCBI BLASTP tool. Multiple alignments were performed using Clustal Omega (http://www.ebi.ac.uk). The NCBI conserved-domain database (CDD), PFAM (http://pfam.xfam.org), and Interpro (http://www.ebi.ac.uk/interpro/) were searched for the identification of functional domains and active site residues. Subcellular localization of proteins was predicted with PSORTb (http://www.psort.org/psortb/) and MycoSub (27). Secondary structures were predicted using PredictProtein (https://www.predictprotein.org).

### Statistical analysis.

An unpaired *t* test was performed using the GraphPad InStat program (GraphPad Software, San Diego, CA) and *P* values of <0.05 were considered statistically significant.

## RESULTS AND DISCUSSION

### *In silico* analysis of M. leprae putative cholesterol metabolism genes.

M. leprae possesses a genome with less than one-third of the functional genes annotated in the M. tuberculosis genome (28), thus retaining what is considered the smallest set of genes necessary for mycobacterial pathogenicity. The annotated M. tuberculosis genome contains approximately 250 genes involved in lipid and fatty acid metabolism, and transcriptional profiling of M. tuberculosis cultured with and without cholesterol identified over 200 genes regulated in response to cholesterol (20). Since intracellular M. leprae survival depends on cholesterol (12), we performed an *in silico* search for M. leprae orthologs of the M. tuberculosis enzymes/proteins associated with cholesterol metabolism. From the 139 M. tuberculosis gene products associated with cholesterol metabolism (21), only 11 were encoded in the M. leprae genome (Table 1). Out of this list, 3β-HSD was likely the only gene product dedicated exclusively to steroid metabolism. The 3β-HSD enzyme of M. tuberculosis has been biochemically validated and shown to oxidize cholesterol to cholestenone, the first step of ring degradation (29) (Fig. 1A). Most of the remaining 10 M. leprae gene products were potential enzymes involved in the beta oxidation cycle, predicted to participate in the degradation of both the cholesterol side chain and fatty acids (21).

**TABLE 1:** Mycobacterium leprae genes exhibiting high similarity to M. tuberculosis genes involved in cholesterol metabolism

M. leprae locus	Gene	Protein description^*d*^	M. tuberculosis locus	% similarity	% coverage
ML0348	*ml0348*	Possible coenzyme F_420_-dependent oxidoreductase	Rv3520c^*a*^	87	97
ML0354	*ilvX*	Putative acetohydroxyacid synthase I large subunit	Rv3509c^*b*^	87	100
ML0559	*ribA*	Putative GTP cyclohydrolase II/3,4-dihydroxy-2-butanone-4-phosphate synthase	Rv1940^*b*^	96	97
ML0660	*fadE23*	Putative acyl-CoA dehydrogenase	Rv3140^*a*^	94	100
ML0661	*fadE24*	Putative acyl-CoA dehydrogenase	Rv3139^*a*^	90	99
ML1051	*xclC (fadD36)*^*e*^	Acyl-CoA synthase	Rv1193^*b*^	88	100
ML1158	*fadA4*	Possible acetyl-CoA C-acetyltransferase	Rv1323^*b*^	93	98
ML1942	*hsd*	Probable cholesterol dehydrogenase	Rv1106c^*c*^	85	99
ML2401	*echA9*	Putative enoyl-CoA hydratase/isomerase	Rv1071c^*a*^	85	100
ML2461	*fadB2*	3-Hydroxyacyl-CoA dehydrogenase	Rv0468^*c*^	94	100
ML2563	*fadE5*	Acyl-CoA dehydrogenase	Rv0244c^*b*^	94	100

a Biochemically predicted.

b Computationally annotated.

c Biochemically validated.

d CoA, coenzyme A.

e *xclC* is alternatively designated *fadD36*.

The remaining set of enzymes involved in the sterol ring and side chain degradation located in the “*cho* island” of M. tuberculosis were not found in M. leprae genome or were identified as pseudogenes. Also, the *mce4* operon, which codes for an active transport system for sterol lipids in mycobacteria, including cholesterol (30), is absent in M. leprae. Taken together, the results of this *in silico* analysis suggest that M. leprae is able to oxidize cholesterol to cholestenone but unable to further degrade its sterol rings and side chain.

### M. leprae is unable to utilize cholesterol as a nutrition source.

The capacity of M. leprae to metabolize cholesterol was further investigated by incubating suspensions of freshly isolated athymic *nu/nu* mouse-derived M. leprae with radiolabeled cholesterol as the sole carbon source. Both [4-^14^C]cholesterol and [26-^14^C]cholesterol, respectively, were used to access the capacity of M. leprae to degrade the sterol ring and the side chain of cholesterol. As a positive control, M. leprae was incubated with [1-^14^C]palmitic acid, the carbon source used in radiorespirometry assays to measure bacterial viability (31). Live M. tuberculosis also served as a positive control, and heat-inactivated M. leprae or M. tuberculosis was used as a negative control. M. leprae cells were incubated for 4 days with the above-mentioned radiolabeled substrates, and the amount of radioactivity incorporated into the bacterial cells was measured. As shown in Fig. 1B, both palmitic acid and cholesterol were incorporated by M. leprae. Only live, not heat-inactivated, bacteria were able to accumulate palmitic acid. In contrast, heat-inactivated M. leprae incorporated cholesterol to a higher level than the live cells. Exogenous cholesterol incorporation into M. leprae was observed as early as 30 min and reached a maximum level at 24 h of incubation. Identical incorporation kinetics were observed with live and heat-inactivated bacteria (data not shown). Since M. leprae has lost the operon (*mce4*) encoding the active transport system for sterols, the observed cholesterol incorporation probably occurs at the cell envelope level through interaction of cholesterol with cell wall components. An explanation for the higher levels of cholesterol incorporation by heat-inactivated M. leprae could be alteration of the cell wall architecture by heat, resulting in the exposure of sites with high-affinity binding of cholesterol. A potential binding site for cholesterol in M. leprae could be the mycolic acids, a major constituent of the mycobacterial cell wall. According to Benadie et al. (32), the mycolic acids mimic the structure of cholesterol and exhibit an affinity for it, being able to interact with cholesterol. These observations could explain the cholesterol incorporation even by heat-inactivated bacteria.

Next, the capacity of M. leprae to oxidize [4-^14^C]cholesterol or [26-^14^C]cholesterol, generating ^14^CO_2_, was measured by a radiorespirometry assay. Moreover, potential utilization of cholesterol for the biosynthesis of bacterial lipids was assessed by extraction of M. leprae lipids and analysis by TLC. As shown in Fig. 1C, live but not heat-inactivated M. leprae oxidized [1-^14^C]palmitic acid, producing ^14^CO_2_. In contrast, increased levels of ^14^CO_2_ production above the background were not observed in the presence of [4-^14^C]cholesterol or [26-^14^C]cholesterol (Fig. 1C). Palmitic acid-derived carbon was also efficiently incorporated into M. leprae lipids, including the species-specific PGL-I, after 4 days of incubation (Fig. 1D). However, when incubated with [4-^14^C]cholesterol or [26-^14^C]cholesterol, M. leprae catalyzed the formation of only one ^14^C-labeled TLC spot in addition to cholesterol. This product migrated with a retardation factor (Rf) consistent with that of cholestenone. Parallel assays were conducted with M. tuberculosis as a control using identical protocols, but with shorter periods of incubation (24 and 72 h). As reported previously, M. tuberculosis was able to utilize cholesterol (18) as well as palmitic acid as a source of energy and carbon (see Fig. S1 in the supplemental material). Altogether, the results generated confirmed the *in silico* prediction that M. leprae is unable to utilize both the sterol rings and the side chain of cholesterol as nutritional sources.

### M. leprae oxidizes cholesterol to cholestenone.

**FIG 2:**
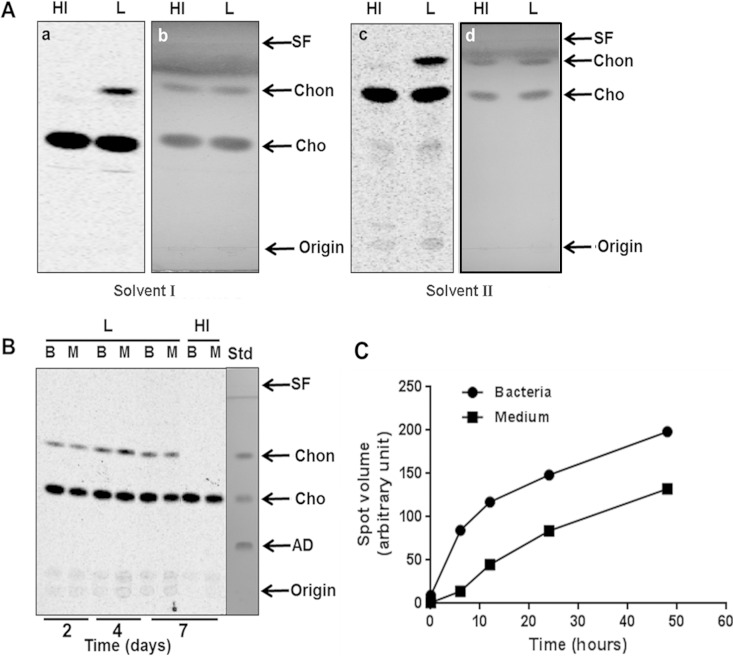
M. leprae oxidizes cholesterol to cholestenone. (A) Comigration on TLC of radiolabeled compounds extracted from M. leprae with cholesterol and cholestenone (a and c). Cholesterol and cholestenone standards (2 μg of each) were used to spike M. leprae lipid extracts and resolved by TLC with solvent I (chloroform-methanol-acetic acid [95:4:1]) or solvent II (chloroform-methanol-ammonium hydroxide [95:4:1]). Cold standards were visualized by spraying the same plates with a 5% phosphomolydbic acid spray and heating them with a heat gun until the bands were visible (b and d). HI, heat inactivated; L, live. (B) Evidence of cholestenone in the spent medium. Bacilli were incubated with [4-^14^C]cholesterol for different time periods at 33°C and separated from spent medium by centrifugation followed by extensive washing with PBS-tyloxapol. Washed bacilli (lanes B) as well as spent medium (lanes M) were extracted with chloroform-methanol (2:1), and the resulting lipids were analyzed by TLC using chloroform-ammonium acetate (97:3) as the running solvent. All TLC plates were exposed to the phosphor screen and radiolabeled compounds were visualized by PhosphorImager analysis. Standards were visualized by spraying the TLC plates with anisaldehyde solution and heating. SF, solvent front; Chon, cholestenone; Cho, cholesterol; AD, androstenedione. (C) Time course of M. leprae cholestenone production and secretion. M. leprae was incubated with [4-^14^C]cholesterol for 0, 6, 12, 24, and 48 h at 33°C. Total lipid extraction and TLC analysis from bacteria and spent medium were performed as described. Densitometry analysis of the chromatogram was performed using the Image QuantTL program. Data are representative of two independent experiments.

To confirm the identity of the product generated from exogenous cholesterol by M. leprae, unlabeled cholesterol and cholestenone standards were spiked into live and heat-inactivated M. leprae radiolabeled lipid extracts. The spiked samples were analyzed by TLC using both acidic (Fig. 2A, solvent I) and basic (Fig. 2A, solvent II) solvent conditions. As shown in Fig. 2A and Table 2, the Rf of the lipid produced by M. leprae from [4-^14^C]cholesterol corresponded to the cholestenone standard. Interestingly, labeled cholestenone was readily extracted from spent medium as well as cells at 2 to 7 days of incubation, as shown in Fig. 2B. Androstenedione (AD), the steroid intermediate generated after the complete degradation of the cholesterol side chain, was not detected in any sample. In concordance with the rapid cholesterol incorporation into the bacterial cells, cholestenone generation was observed as early as 6 h after incubation with cholesterol (Fig. 2C). This rapid conversion of cholesterol to cholestenone, in the likely absence of a cholesterol transport mechanism, suggests that cholestenone formation occurs at the bacterial cell surface.

**TABLE 2:** Retardation factor (Rf) values of the radiolabeled compound produced by M. leprae incubated with [4-^14^C]cholesterol

Substance	Rf value for solvent^*a*^							
	I				II			
	Standard		Labeled sample		Standard		Labeled sample	
	HI	L	HI	L	HI	L	HI	L
Cholesterol	0.52	0.51	0.52	0.51	0.72	0.72	0.74	0.74
Cholestenone	0.77	0.78		0.78	0.90	0.90		0.91

a Solvent I, chloroform-methanol-acetic acid (95:4:1); solvent II, chloroform-methanol-ammonium hydroxide (95:4:1); L, live; HI, heat inactivated.

### Prediction of the M. leprae cholesterol oxidase.

**FIG 3:**
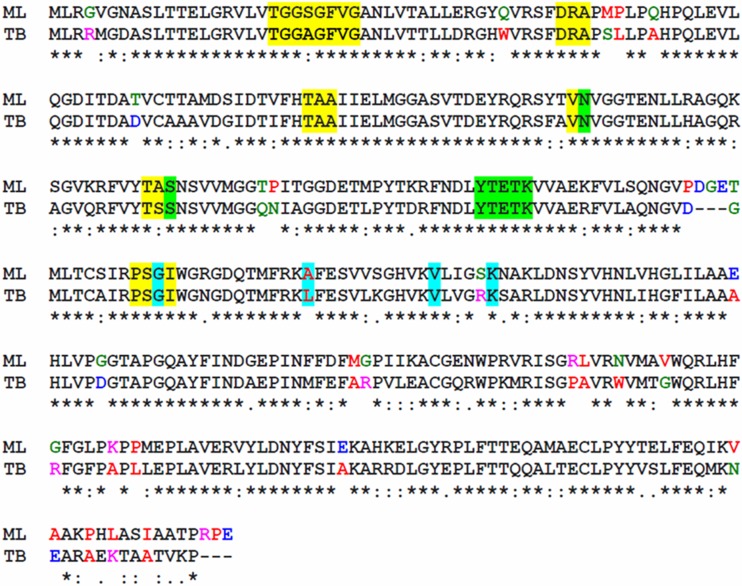
3β-Hydroxysteroid dehydrogenase protein sequences from M. leprae (ML1942) and M. tuberculosis (Rv1106c). Asterisks indicate positions which have identical amino acid residues, colons indicate conserved amino acid substitution between groups with strongly similar properties, and periods indicate amino acid conservation between groups with weakly similar amino acid properties, according to Clustal software. Yellow highlighting indicates the NAD binding motif, green highlighting indicates the active-site residues, and blue highlighting indictates additional substrate binding residues. Nonconserved substitutions are indicated by colored amino acid residues, according to the Clustal color code: red, small and hydrophobic; blue, acidic; magenta, basic; green, containing hydroxyl, sulfhydryl, and amine. ML, M. leprae; TB, M. tuberculosis.

The first reaction of ring metabolism is the oxidation and isomerization of cholesterol to form cholestenone (Fig. 1A). In bacteria, this process is catalyzed by either 3β-HSD or cholesterol oxidase. Although these enzymes utilize distinct reaction mechanisms, they catalyze the same transformation, and both can be found within the genomes of steroid-utilizing bacteria (21). The capacity of M. leprae to oxidize cholesterol to cholestenone was expected based on the annotation of an intact *hsd* gene (*ml1942*). The M. leprae 3β-HSD ortholog is 75% identical and 85% similar to the M. tuberculosis 3β-HSD (Rv1106c) (Table 1), whose cholesterol oxidation activity was experimentally confirmed (29). ML1942 contains a conserved NAD binding motif, as well as a conserved active site and substrate binding residues (Fig. 3). The nonconserved substitutions between M. leprae and M. tuberculosis proteins do not result in significant changes in the predicted secondary structure (data not shown). Thus, the bioinformatics of ML1942 provides evidence that this protein functions as a 3β-HSD that would allow M. leprae to convert cholesterol to cholestenone.

A previous proteomics study showed the presence of the enzyme associated with the cell wall of M. leprae (33). This subcellular localization is consistent with the current data that demonstrated the absence of the cholesterol import machinery in M. leprae and rapid accumulation of cholestenone in the spent culture medium, supporting the hypothesis that cholesterol transformation to cholestenone occurs at the bacterial surface.

Besides the *hsd* gene, the genomes of M. leprae and M. tuberculosis encode annotated ChoD cholesterol oxidase proteins (ML0389 and Rv3409c, respectively). The M. leprae ChoD possesses high sequence similarity to the M. tuberculosis ChoD (see Fig. S2 in the supplemental material). However, alignment of these two sequences to a functional ChoD (PDB ID 1B4V) from Streptomyces sp. revealed an absence of amino acid residues composing the well-defined FAD binding and active sites required for cholesterol oxidase activity (see Fig. S2 in the supplemental material). In addition, the two loop regions of Streptomyces sp. and Brevibacterium sp. cholesterol oxidases involved in positioning the substrate in the active site are weakly conserved in putative mycobacterial ChoD proteins. Furthermore, ChoD shows a low level of amino acid identity (∼24%) with cholesterol oxidases from other bacteria as well as other glucose-methanol-choline (GMC) oxidoreductase superfamily members; only the FAD binding region is conserved (34, 35). Moreover, a recent study by Gao and Sampson (36) suggests that Rv3409c controls acetylation of cell surface glycopeptidolipids, which affects activation of the innate immune system. Experimental evidence also indicates that the M. tuberculosis ChoD does not catalyze the transformation of cholesterol to cholestenone (37); in addition, an M. tuberculosis strain with a deletion of the *hsd* gene was incapable of oxidizing cholesterol (29, 37). This provides further evidence that 3β-HSD is the sole cholesterol-oxidizing enzyme in M. tuberculosis and that the ChoD of M. leprae and M. tuberculosis may have a function other than cholesterol oxidation.

### *In vivo* production of cholestenone by M. leprae.

**FIG 4:**
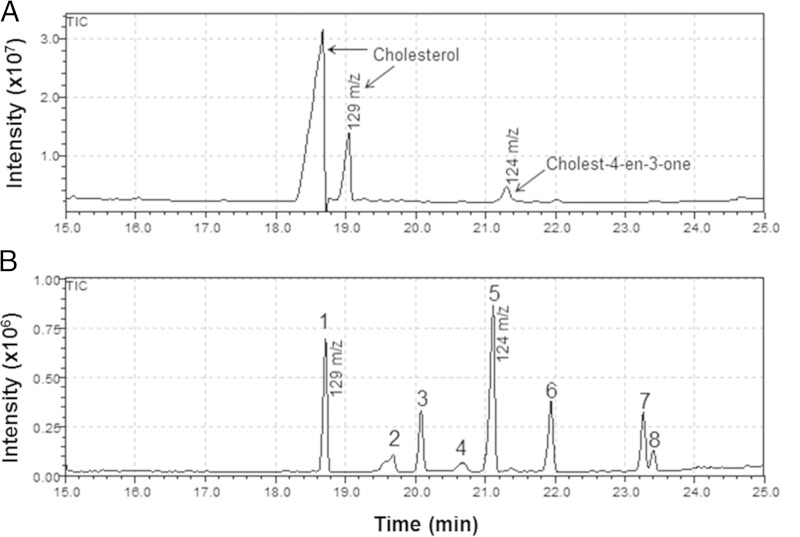
Cholestenone is produced *in vivo* by M. leprae. (A) GC-MS chromatogram of bis(trimethylsilyl)trifluoroacetamide (BSTFA)-trimethylchlorosilane (TMCS)-derived extracts of M. leprae; (B) steroid standards mix. M. leprae was isolated from footpads of infected mice and submitted to total lipids extracted with chloroform-methanol (2:1). Dried lipids were saponified with 25% alcoholic potassium hydroxide solution, extracted with heptane, and derivatized with BSTFA-TMCS (99:1) and pyridine. Standards: 1, cholesterol; 2, cholesta-5,7-dien-3-ol; 3, cholesta-8,24-dien-3-ol; 4, ergosta-5,7,22-trien-3-ol; 5, cholest-4-en-3-one; 6, stigmasterol; 7, lanosta-8,24-dien-3-ol; 8, β-sitosterol.

The M. tuberculosis 3β-HSD recombinant enzyme was shown to also oxidize other 3-hydroxysterols, such as pregnenolone and dehydroepiandrosterone, to their respective 3-keto-4-ene products (29). Thus, we investigated whether the *in vitro* transformation of cholesterol to cholestenone by M. leprae occurs during *in vivo* infection. M. leprae isolated from footpads of infected mice was subjected to lipid extraction followed by GC-MS analysis. Two peaks of cholesterol were detected, the first corresponding to nonsilylated cholesterol (Fig. 4A). This observation correlated with the *in vitro* data that demonstrated the accumulation of cholesterol on the cell surface of M. leprae. A smaller peak with a retention time and mass identical (Fig. 4A; also, see Fig. S3 in the supplemental material) to those of cholestenone was also detected in the footpad-derived M. leprae. Since trace amounts of cholestenone can be found in mammalian tissue (38), the possibility that the detected cholestenone was derived from contaminating host tissue was assessed by analyzing lipids directly extracted from uninfected mouse footpad tissue. As shown in Fig. S4A in the supplemental material, no cholestenone was detected in uninfected tissue, indicating that the cholestenone found in M. leprae purified from the mouse footpad was truly a product of *in vivo* bacterial metabolism.

The retention of the *hsd* gene in the genome of M. leprae, despite the reductive evolution of M. leprae, and the observed production of cholestenone by M. leprae in infected tissue suggests a role for sterol-oxidizing activity in bacterial pathogenesis. We recently revealed that M. leprae infection induces the expression and activation of sterol regulatory element-binding protein (SREBP), a transcription factor that regulates the biosynthesis and uptake of cholesterol (12, 39). Liver X receptor (LXR), another important transcription factor, provides a regulatory system for the elimination of excess cholesterol (40). The activities of the SREBP and LXR pathways are regulated by several sterols and oxysterols that also influence innate and adaptive immune responses in chronic diseases (41). One of the most abundant oxysterols is 27-hydroxycholesterol (27HC), which is generated by the enzyme sterol 27-hydroxylase (CYP27A1) (42, 43). *In vitro* studies have shown that cholestenone is hydroxylated at a much higher rate than corresponding sterols with a 3-hydroxy-Δ^5^ structure (44). Thus, there is a possibility that the host cell CYP27A1 can oxidize cholestenone produced by M. leprae. Furthermore, recent data have suggested that an excess of cholestenone affects host cell membrane functionality (45–48). Therefore, we propose that cholestenone production by M. leprae might modulate host cell functions to facilitate M. leprae invasion and persistence in those cells.

Finally, the accumulation of cholesterol *per se* can play an important role in bacterial pathogenesis. Recently, different roles for cholesterol during microbial infection as well as in cell processes have been described. Cholesterol increases Helicobacter pylori resistance to several antibiotics, such as tetracycline and clarithromycin, due to its incorporation in the bacterial membrane (49). It has been reported that M. tuberculosis incorporates cholesterol into the cell wall, leading to decreased rifampin uptake (19). This phenomenon could explain the enhanced mycobacterial killing during the combined treatment of M. leprae- and M. tuberculosis-infected macrophages with atorvastatin and rifampin (15). Furthermore, as mentioned before, cholesterol may interact with mycolic acids in the cell wall (32), leading to an even more decreased cell wall permeability, since cholesterol has major implications in membrane fluidity and rigidity. Considering that rifampin is able to cross the cell wall due to its hydrophobicity (50), any alterations in the cell wall permeability can directly impact rifampin on uptake, as observed by Brzostek et al. (19). In addition, cholesterol accumulation has been implicated in inhibiting phagosome fusion with lysosomes (51), and reports showed that M. leprae inhibits phagolysosome fusion, which contributes to its survival inside the host cell (52). Taken together, this evidence supports the idea that cholesterol accumulation influences, in multiple ways, host-pathogen interactions that might contribute to infection persistence.

In conclusion, our data indicate that M. leprae lost the capacity to catabolize the sterol rings and the side chain of cholesterol, preserving only the capacity to oxidize it to cholestenone, the first step of cholesterol degradation. It was also shown that cholesterol is likely incorporated into the cell envelope by a passive mechanism, since M. leprae has lost the *mce4* operon responsible for coding the active transport system in mycobacteria devoted to sterols. Interestingly, M. tuberculosis strains with mutations in *mce4* also retain some ability to incorporate cholesterol. This residual cholesterol uptake was suggested to be due to another, less efficient import system or to a passive diffusion into the cell (18). Transformation of cholesterol to cholestenone was observed to occur both *in vitro* and *in vivo* and most likely occurs at the bacterial cell surface catalyzed by the enzyme 3β-HSD. Our study underscores the complexity of sterol metabolism and that its importance for mycobacterial pathogenesis extends well beyond its utilization as a source of nutrition. This is also evident in the context of M. tuberculosis infection, where cholesterol accumulation in the host cell but not its degradation seems to be important for bacterial survival during the active phase of infection when multiple other carbon sources are available for sustaining bacterial intracellular growth (21, 53, 54). Thus, a future challenge is to understand the biochemical basis of the role of cholesterol, including its oxidation step, in M. leprae pathogenesis. Further elucidation of cholesterol metabolism in the host cell during M. leprae infection will establish the mechanism by which this lipid supports M. leprae intracellular survival and will open new avenues for novel leprosy therapies.

## Supplementary Material

Supplemental material
