# Supplementary material for: The Essential Role of Cholesterol Metabolism in the Intracellular Survival of Mycobacterium leprae Is Not Coupled to Central Carbon Metabolism and Energy Production
Source: J Bacteriol. 2015 Oct 28;197(23):3698–707. doi: 10.1128/JB.00625-15 (PMC4626898; doi:10.1128/JB.00625-15)
Supplement: Supplemental material [file supp_197_23_3698__index.html]

Supplemental material 

# The Essential Role of Cholesterol Metabolism in the Intracellular Survival of Mycobacterium leprae Is Not Coupled to Central Carbon Metabolism and Energy Production

## Supplemental material

- Supplemental file 1 -

  Fig. S1, *M. tuberculosis* utilizes [4-14C]cholesterol

  Fig. S2, putative features of *M. leprae* ChoD protein

  Fig. S3, mass spectrum of the peak correspondent to cholestenone

  Fig. S4, GC-MS analysis

  PDF, 4.4M
