# Supplementary material for: The Essential Role of Cholesterol Metabolism in the Intracellular Survival of Mycobacterium leprae Is Not Coupled to Central Carbon Metabolism and Energy Production
Source: J Bacteriol. 2015 Oct 28;197(23):3698–707. doi: 10.1128/JB.00625-15 (PMC4626898; doi:10.1128/JB.00625-15)
Supplement: Supplemental material [file JB.00625-15_zjb999093831so1.pdf]

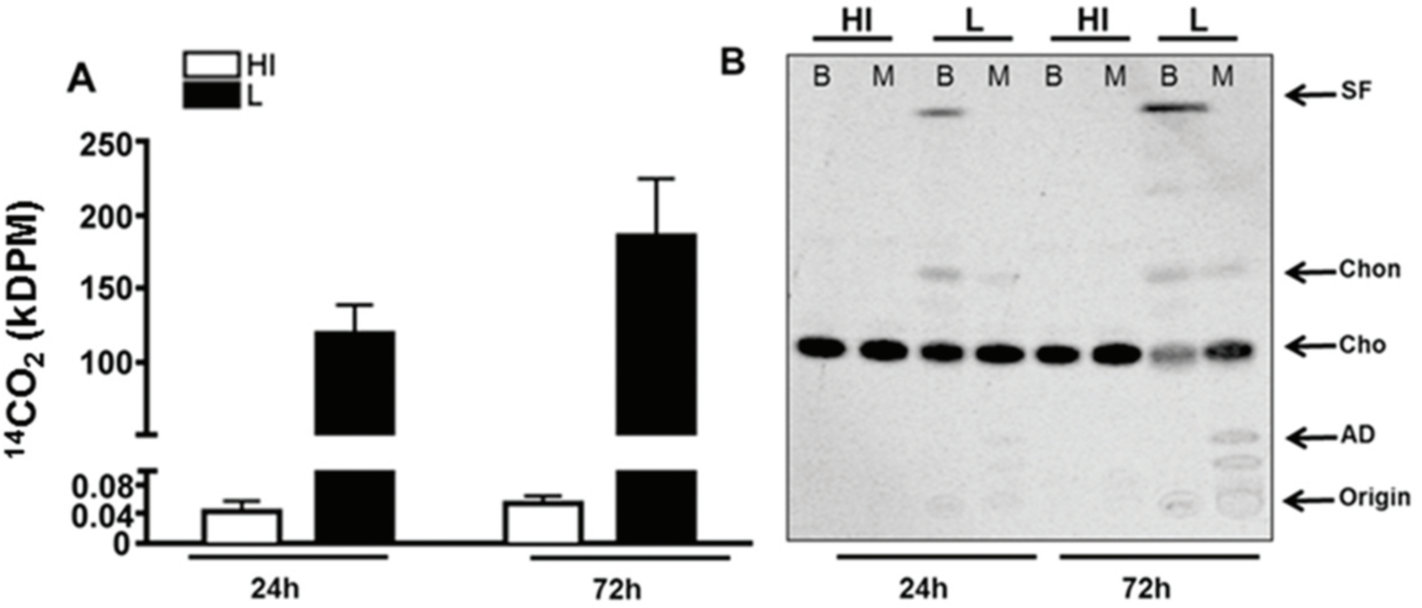

Fig. S1. ***M. tuberculosis*** **utilizes [4-^{14}C]cholesterol as energy and carbon source.** A) *M. tuberculosis* (1.6 OD/ml, live (L) or heat inactivated (HI) at 100°C/15 min) was incubated with 1 µCi/ml [4-^{14}C]Cholesterol (50 mCi/mmol) for 24 and 72 h at 37 °C. Labeled CO_2 trapped on strips was determined and expressed in DPM. Data are representative of two independent experiments duplicate. B) *M. tuberculosis* cells were washed, extracted with chloroform/methanol 2:1 and analyzed by TLC using chloroform/ammonium acetate (97:3) as solvent system. Radiolabeled lipids were detected by PhosphorImager. B, bacteria cells; M, spent medium; SF, solvent front; Chon, cholestenone; Cho, cholesterol; AD, androstenedione; SF, solvent front.

```
1B4V  --GYVPAVVIGTGYGAAVSALRLGEAGVQTLMLEMGQLWNQPGPDGNIFCGMLNPDKRSS
ML    MKPDYDVLIIGSGFGGSVSALRLTEKGYRVGVLEAGRRFADEDFA---------------
TB    MKPDYDVLIIGSGFGGSVTALRLTEKGYRVGVLEAGRRFSDEEFA---------------
            .::**:*:*.:*:**** * * :. :** *: : :

1B4V  WFKNRTEAPLGSFLWLDVVNRNIDPYAGVLDRVNYDQMSVYVGRGVGGGSLVNGGMAVEP
ML    ----KTSWDLRKFLWAPKLGC-----YGIQRIHLLRNVMILAGAGVGGGSLNYANTLYVP
TB    ----KTSWDLRKFLWAPRLGC-----YGIQRIHPLRNVMILAGAGVGGGSLNYANTLYVP
          :*.  * .***   :        *:       :: : .* *******   .    *

1B4V  KRSYFEEILP-RVD-SSEMYDRYFPRANSMLRVNHIDTKWFEDTEWYKFARVSREQAGKA
ML    PEPFFANQQWAHITDWHSELAPHYDQAQRMLGVV-------CNPTFTDADRILKEVVDEM
TB    PEPFFADQQWSHITDWRGELMPHYQQAQRMLGVV-------QNPTFTDADRIVKEVADEM
       . :* :    ::          :: :*: ** *          :  :  .   *: :* . :

1B4V  GLGTVFVPNVYDFGYMQR--EAAGE------------VPKSALA-TEVIYGNNHG-KQSL
ML    GFGDTFVPTPVGVFFGPDGTQTPGRTVADPYFGGVGPVRTGCLECGCCMTGCRHGAKNTL
TB    GFGDTWVPTPVGVFFGPDGTKTPGKTVPDPYFGGAGPARTGCLECGCCMTGCRHGAKNTL
      *:* .:**.    . :      :: *.              . ...*      : * .** *::*

1B4V  DKTYLAAALGTGKVTIQTLHQVKTIRQTKDGGYALTVEQKDTDGKLL--ATKEISCRYLF
ML    VKNYLGLAESAGAQVI-PMTTVKGFELRSEGLWEVHTV---RTGSWLRRGRRTFTAAHLL
TB    VKNYLGLAESAGAQVI-PMTTVKGFERRSDGLWEVRTV---RTGSWLRRDRRTFTATQLV
       *.**. * .:*   .*  :   ** :.   .:* : : .        *. *     : ::.  *.

1B4V  LGAGSLGSTELLVRARDTGTLPNLNSEVGAGWGPNGNIMTARANHMWNPTGAH------Q
ML    LAAGTWGTQRLLFRMRDQGKLPRLSQRLGVLTRTNSESIVGAGTLNVMPDRDLTHGVAIT
TB    LAAGTWGTQHLLFKMRDRGRLPGLSKRLGVLTRTNSESIVGAATLKVNPDLDLTHGVAIT
      *.**: *: .**.: ** * ** *...:*.     *.: :.. ..     *

1B4V  SSI--------PAL----GIDAWDNSDSSVFAEIAPMPAGLETWVSLYLAITKNPQRGTF
ML    SSIHPTSDTHIEPIRYGKGSNAMGLLQTLMTDGPGPEGTDVPRWRQLLHQASEDPRRMLR
TB    SSIHPTADTHIEPVRYGKGSNAMGLLQTLMTDGSGPQGTDVPRWRQLLQTASQDPRGTIR
      ***            :    * :*     :: :      .*  : :  * .*     :::*:

1B4V  VYDA------------------------ATDRAKLNWTRDQNAPA------VN----AAK
ML    LINPRRWSERTVIALVMQHLDNSITTFTKRGKLGIRWYSSKQGNGEPNPSWIPIGNEVTR
TB    MLNPRQWSERTVIALVMQHLDNSITTFTKRGKLGIRWYSSKQGHGEPNPTWIPIGNQVTR
      : :                                 :  :.*  .::. .       :     .::

1B4V  ALFDRINKANGTIYRYDLFGTQLKAFADDFCYHPLGGCVLGKA-----TDDYGRVAGYKN
ML    RLAAKIDGVAG--------GTWGELFNIPLTAHFLGGAVIGDNAEHGVIDPYHRVYGYPT
TB    RIAAKIDGVAG--------GTWGELFNIPLTAHFLGGAVIGDDPEHGVIDPYHRVYGYPT
       :  :*: . *         **  : *    :  * ***.*:*.         * * ** ** .

1B4V  LYVTDGSLIPGSVGVNPFVTITALAERNVERIIKQDVTAS--------------------
ML    LYVVDGAAISANLGVNPSLSIAAQAERAASLWPNKGQHDQRPRQGESYRRLAPIAPDHPV
TB    LYVVDGAAISANLGVNPSLSIAAQAERAASLWPNKGETDRRPPQGEPYRRLAPIQPAHPV
      ***.**: * ..:**** ::*:* ***  ..   ::

1B4V  ---------------------
ML    VPAEALGALRWL---------
TB    VPADAPGALRWLPIDPVSNAG
```

S2: Putative features of *M. leprae* ChoD protein. Alignment of primary sequences of a functional cholesterol oxidase (1B4V, *Streptomyces sp*.) and putative ChoD proteins from *M. leprae* (ML) and *M. tuberculosis* (TB). Asterisks (*) indicate positions, which have a single, fully conserved amino acid residue. Colons (:) indicate conservation between groups of strongly similar properties and periods (.) indicate conservation between groups of amino acids with weakly similar properties, according to Clustal software. Amino acids and sequences shaded in grey are not fully conserved in ML and TB proteins. FAD binding motifs are indicated in yellow, active site residues in highlighted in green and the loop regions important for substrate specificity are coloured in blue.

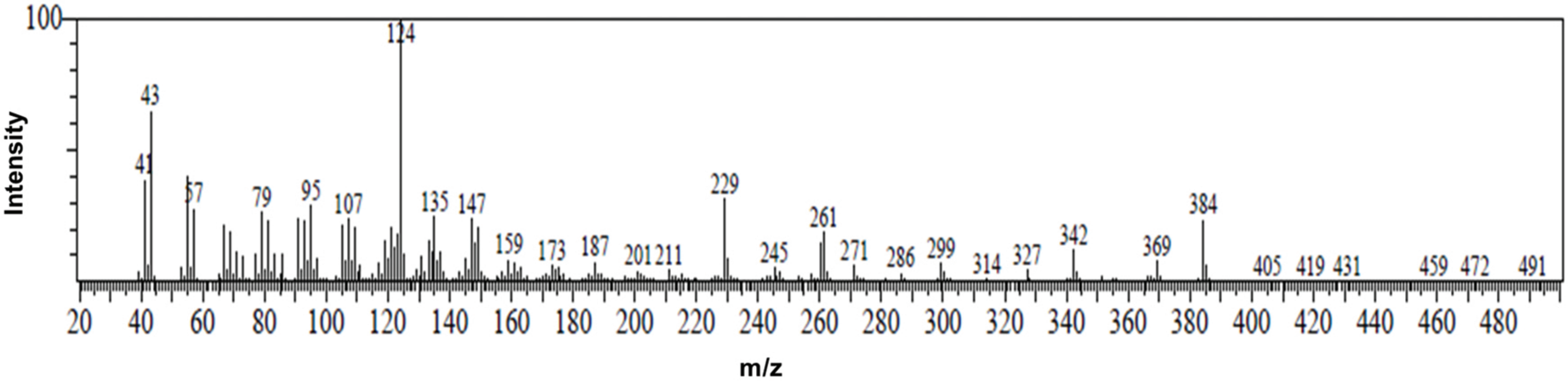

Fig. S3. Mass spectrum of the peak correspondent to cholestenone present on lipid extract of *nude* mouse derived-*M. leprae.*

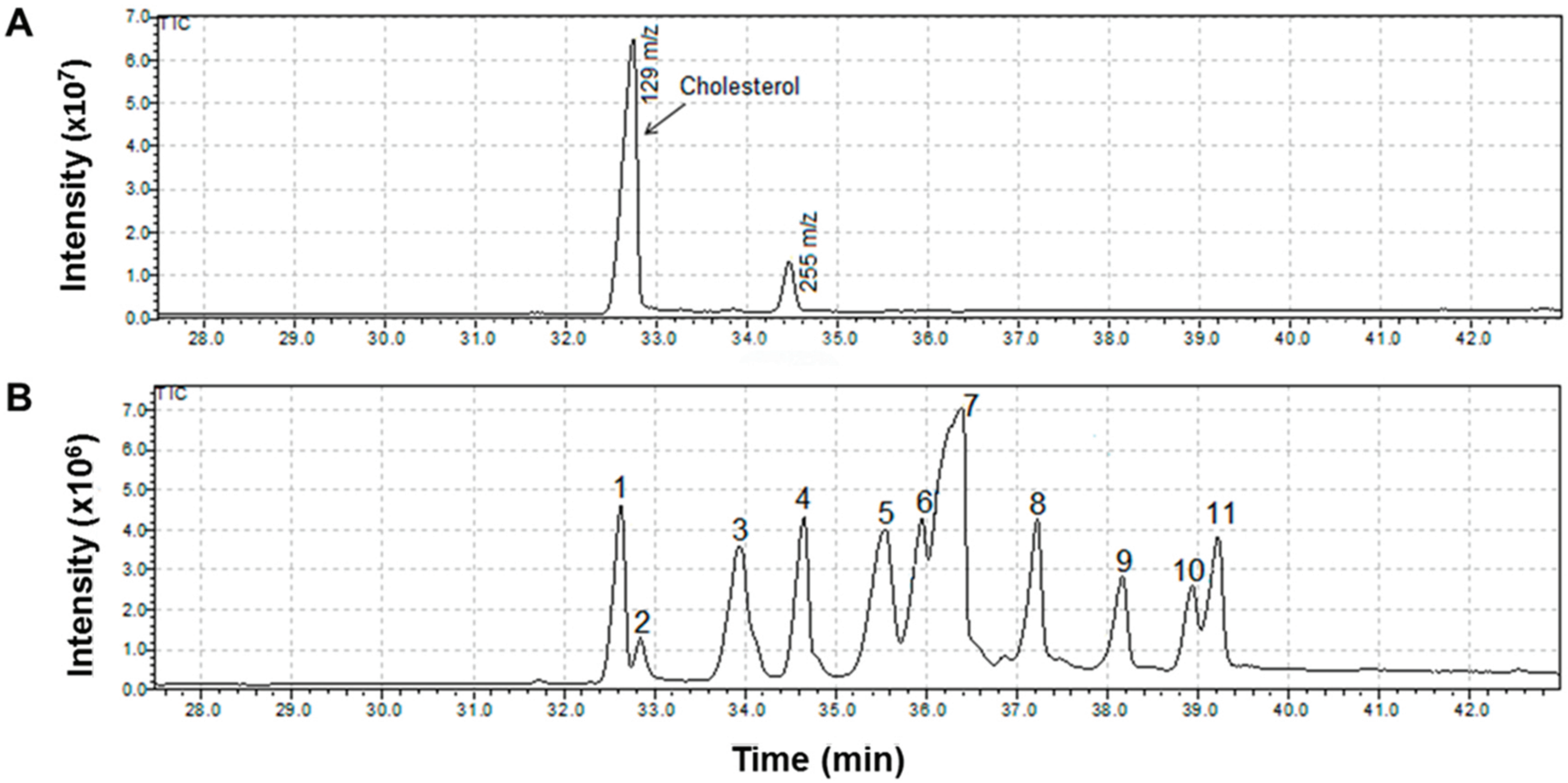

Fig. S4. GC-MS analysis of non-infected *nude* mouse footpad lipid extract A) GC–MS chromatogram of Bis(trimethylsilyl)trifluoroacetamide (BSTFA) - trimethylchlorosilane (TMCS)-derived extracts of non-infected *nude* mouse footpad and **B**) steroid standards mix. Standards: 1, Cholesterol; 2, ergosta-7,22-dien-3-ol; 3, cholesta-5,7-dien-3-ol; 4, cholesta-8,24-dien-3-ol; 5, ergosta-5,7,22-trien-3-ol; 6, cholest-4-en-3-one; 7, ergost-8(14)-en-3-ol; 8, stigmasterol; 9, ergost-7-ene; 10, lanosta-8,24-dien-3-ol; 11, β-Sitosterol
